# Review of Navigation Assistive Tools and Technologies for the Visually Impaired

**DOI:** 10.3390/s22207888

**Published:** 2022-10-17

**Authors:** Mohamed Dhiaeddine Messaoudi, Bob-Antoine J. Menelas, Hamid Mcheick

**Affiliations:** Department of Computer Sciences and Mathematics, University of Quebec at Chicoutimi, 555 Blv Universite, Chicoutimi, QC G7H 2B1, Canada

**Keywords:** navigation method, speech and voice recognition, deep learning, blind people, visually impaired

## Abstract

The visually impaired suffer greatly while moving from one place to another. They face challenges in going outdoors and in protecting themselves from moving and stationary objects, and they also lack confidence due to restricted mobility. Due to the recent rapid rise in the number of visually impaired persons, the development of assistive devices has emerged as a significant research field. This review study introduces several techniques to help the visually impaired with their mobility and presents the state-of-the-art of recent assistive technologies that facilitate their everyday life. It also analyses comprehensive multiple mobility assistive technologies for indoor and outdoor environments and describes the different location and feedback methods for the visually impaired using assistive tools based on recent technologies. The navigation tools used for the visually impaired are discussed in detail in subsequent sections. Finally, a detailed analysis of various methods is also carried out, with future recommendations.

## 1. Introduction

Vision-based methods are a specialized method of allowing people to observe and infer knowledge elicited from the environment. The environment may not only be restricted indoors but may extend outdoors as well. A visually impaired person usually faces several challenges for safe and independent movement both indoors and outdoors. These challenges may be more considerable while navigating through an outdoor environment if a person is unfamiliar with the new background and context due to reduced and contracted vision. To overcome mobility restrictions such as walking, shopping, playing, or moving in an outdoor environment, many assistive tools and technologies have been proposed as wearable devices, allowing users to interact with the environment without triggering any risk. These assistive tools improve the quality of life of the visually impaired and make them sufficiently capable to navigate indoors and outdoors [1].

Statistics published by the World Health Organization (WHO) have revealed that one-sixth of the world population is visually impaired, and that figure is sharply increasing [2]. Becoming visually impaired or blind does not imply that a person cannot travel to and from locations at any time they desire. These individuals with visual deficits and diseases require support to complete everyday tasks, which include walking and investigating new areas, just as an average person without disabilities does. Insecure and ineffective navigating ranks among the most significant barriers to freedom for visually impaired and blind persons and assisting these visually impaired users is an important research area. Traditionally, guide dogs and white canes have served as travel assistants. However, they can only partially provide independent and safe mobility. Recent advances in technologies, however, have broadened the spectrum of solutions. From solutions based on radars in the mid-20th century to the current Artificial Intelligence (A.I.), emerging assistive techniques have played a vital role in designing wearable devices for the visually impaired. State-of-the-art techniques in these primary wearable assistive devices incorporate Global Systems for GSM mobile communication along with G.P.S. tools and techniques that automatically identify the location of the wearer and transfer that location to their guardian device [3].

Similarly, Electronic Travel Aids are based on sensors such as infrared, ultrasonic, Radio Frequency Identification (RFID), and G.P.S. to perceive the environment, process the information, and detect objects [4]. Further, many persons are unable to play video games because of visual impairments and have restricted accessibility while interacting with video games and taking part in various educational, social, and physical activities [5]. Game-based approaches to training in navigation systems in unfamiliar environments can help the players build a reliable spatial cognitive map of the surroundings, while audio cues processed by sonification can help players recognize objects in the gaming environment. For example, verbal notifications can help players grasp their location and tell them which task must be accomplished. On the other hand, non-verbal cues can indicate the meta-level of knowledge regarding the target object’s location, direction, and distance to build spatial cognitive maps [6].

While considering the popularity of smart devices among the visually impaired, an optimized solution using a smartphone does not yet exist [7,8,9]. This review study addresses various solutions that allow visually impaired users to walk more confidently across a street. Moreover, smartphone-based solutions are extended to both indoor and outdoor environments. However, no efficient solution for a real-time indoor navigation system exists yet either. A comparison of current navigation systems is shown in Table 1, which also allows a subjective evaluation of the technologies based on user needs.

In contrast with the indoor environment, the GPS-based navigation systems consume more power in the outdoor environment than proposed technologies such as Zigbee and Bluetooth [10]. Bluetooth is not comparable with ZigBee due to its power consumption. If an application is operated for an extended period on a battery, e.g., using G.P.S., Bluetooth will not be adequate. Bluetooth design recommends one watt of power consumption. Still, when combined with wireless G.P.S. applications, the power consumption of both Bluetooth and ZigBee is between 10 and 100 milliwatts (mW), which is 100 times less than previous Bluetooth designs [11]. With Wireless Sensor Network (WSN) technology, tracking and navigation have become more accessible and convenient. In fact, WSN has progressed as a dominant field of research. Thus, multiple tools and technologies have been proposed in the literature and incorporated into natural environments to assist visually impaired users. Similarly, several surveys and reviews have summarized state-of-the-art assistive technologies for visually impaired users. These technologies provide a broad spectrum of techniques that could further progress [12,13,14].

Various experiments conducted by researchers used an audio screen linked with N.V.D.A.D.A. screen reading software. The audio screen allowed a blind user to move the fingers, pen, or mouse on the picture shown on the screen and hear information about the part they touched. The user could explore the maps of countries, colors of the rainbow, and cartoon characters. The audio screen allowed the user to hear the description of the text (font, size, and color) The audio screen was further divided into two output modes. The first was “pitch stereo grey”, which was helpful in the description of images, maps, and diagrams. The second one was “HSV Colors,” which described the variations in colors of photographs [15].

Multiple articles were reviewed in detail while formulating this survey article. The obstacle recognition method, feedback methods, and navigation technologies for the visually impaired are briefly explained in Section 2. Different navigation and feedback tools are discussed in Section 3, while Section 4 contains discussions about the papers. Section 5 concludes the research survey, provides future directions for researchers, and presents the pros and cons from the perspective of visually impaired users.

## 2. Navigation Technologies for the Visually Impaired

### 2.1. Location Methods

Several indoor and outdoor orientation and navigation-based solutions exist commercially for the visually impaired, which are also in active research and development. This section briefly explains multiple wireless navigation methods, including ZigBee, Bluetooth, Wi-Fi, and GPS-based ultrasonic sensors. Moreover, several approaches have been applied for utilizing Wi-Fi hotspots for indoor localization and navigation. Wi-Fi-based strategies incorporate Received Signal Strength Indicator (RSSI) localization methods for measuring the signal strength and calculating the geographical location using G.P.S. using Triangulation [16]. The following subsections discus and analyze the processes and their applications.

#### 2.1.1. Triangulation

Almost every direct-sensing technique aims to locate the user by sensing a unique identifier. However, some systems also employ various identifiers and incorporate computational triangulation approaches to determine the user’s location. These methods/techniques determine such a location using triangulation-based sensors installed in specified areas. The tags frequently incorporated for the localization of users include RFID, ultrasound, Bluetooth, and infrared [17].

#### 2.1.2. Trilateration

Trilateration is a method used to measure a point location by utilizing the geometry of triangles, circles, or spheres. The triangulation uses the measurement of the angle to determine location, while trilateration uses the measurement of distance. To provide accurate global surface and geographical locations, Land Surveys and G.P.S. use this method.

### 2.2. Innovation and Localization Technologies

#### 2.2.1. Triangulation Using GPS-Based System

The Global Positioning System (G.P.S.), which operates 27 satellites orbiting the earth, is mainly used for outdoor navigation. The satellites are positioned at any point on the planet, which is covered by at least four satellites at any particular time. Each satellite sends positional information about itself (ephemeris) and all other satellites (almanac). The G.P.S. receiver determines its distance from several satellites using data from the ephemeris that employs the triangulation principle, by which the time the data are sent is compared to the time it is received. Thus, at least three satellites are needed to complete the process. However, more satellites allow better precision. This method requires the G.P.S. receiver to be well exposed to the satellite, which prevents indoor locations.

G.P.S. systems are widely used for outdoor navigation [18]. The main disadvantage of G.P.S. localization is that the G.P.S. signal strongly degrades inside buildings, between tall buildings, or in dense forest areas (such as parks) [19]. G.P.S. is inadequate for visually impaired individuals because of its low precision. It also becomes challenging to use such systems in locations with unknown obstacles. Technologies that use these G.P.S. systems, such as guide canes and Braille signs, are not viable options for visually impaired individuals to find their way in new outdoor environments [20]. A G.P.S. position or location is measured based on distance from other objects or points with known sites. The area of each of the satellites is already known with accuracy, and a G.P.S. receiver measures the distance from an object with the help of the satellites [21].

#### 2.2.2. Triangulation Using the ZigBee-Based System

ZigBee [22] is a wireless navigation technology for the visually impaired that can be used in indoor/outdoor navigation systems. It has high accuracy in location, wide-coverage, simple infrastructure, reduced cost, quick real-time navigation, and lower power consumption. ZigBee Triangulation is based on IEEE 802.15.4, which uses the 868 MHz band in Europe, the 915 MHz band in North America, and the 2.4 GHz band [23].

**Figure 1:**
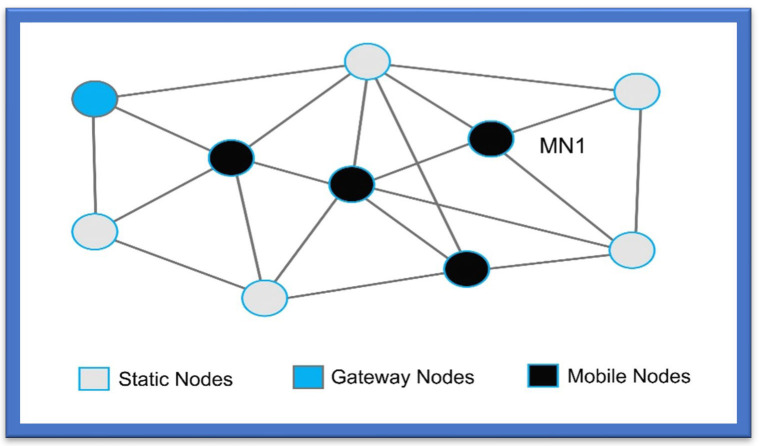
ZigBee Triangulation Mesh Network.

This triangulation process is used to transmit signals at long distances among the devices within the wireless mesh networks as shown in Figure 1. This process is known to have a low data transfer rate, low cost, and short-latency time compared to other Wi-Fi standards. As per the IEEE standards of 802.15.4, “Link quality indication (L.Q.I.)” is taken as the process to specify the link quality and is exploited to derive the “Received Signal Strength (RSS)” [24].

Xiao et al. [25] designed the structure of a multi-sensor-based innovative system that gives a “high monitoring accuracy” for users’ status and position. The ZigBee-based system consists of multiple sensors, such as an ultrasound transmitter and receptor, temperature sensors (LM35), a tilt-compensated compass, “liquid propane gas (Figaro LPM2610)”, and stick and accelerometer. Moreover, “ZigBee wireless protocols” were also used for XM2110 modules. The user’s navigation and tracking were deployed and executed with a “multi-trilateration algorithm (M.T.A.)”. Navigation accuracy came out to be 3.9 cm in a real-time scenario.

#### 2.2.3. Triangulation Using a Bluetooth-Based System

**Figure 2:**
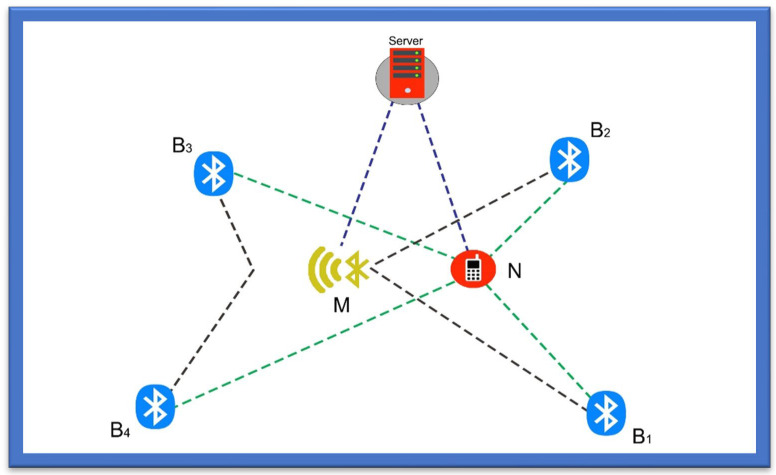
Bluetooth Triangulation.

Another category for wireless systems is based on the Bluetooth Triangulation [26] method, as it is an easy method already deployed in mobile phones, cameras, handheld devices, and gadgets that can be connected over a shorter distance. It is low cost, has a reduced weight and reduced size, and provides power savings. However, Bluetooth can only communicate over a shorter distance and has limitations when used in an application that involves communication over long distances as shown in Figure 2 [27].

The Bluetooth triangulation method was used where Bluetooth module WT-12 was first proposed by Bluegiga; this module is considered Class 2 Bluetooth, with a range of 10 m and an in-built chip antenna [28]. In this study the module was combined with the evaluation board, which was used as an inquiry generator for each Bluetooth receiver. In addition, a G.U.I (Graphical User Interface) was developed to calculate and estimate the RSSI designed to record every value from the register of the Bluetooth Module after the inquiry cycle. Furthermore, M.A.C. addresses of every Bluetooth module with their location on a 15 × 15 grid were also entered before the testing. In the whole process, the inquiry cycle ran 25 times on each spot, for which the coordinates were changed manually after each run [29,30,31].

In a study by Andò et al. [27], the proposed method allows people with disabilities to navigate in an indoor environment. The study measured the system performance based on the W.S.N. system, which enhances the lives of the visually impaired, wheelchair users, the deaf, or persons with other physical disabilities by helping to locate them and their surroundings and navigate them to their destination. The system’s structure comprises a smartphone and wireless technology of Bluetooth and a routing engine for navigating persons, and RSS and WASP for facilitating communication through a smartphone. The Bluetooth-based navigation system showed an efficiency of about 90% in a study carried out with ten users.

#### 2.2.4. Triangulation Using a Wi-Fi-Based System

A Wi-Fi-based navigation [32] system enables device connection anytime, anywhere, at home, work, shopping malls, and in hospitals. It is compatible with different systems, from medical devices to printers and tablets. While using Wi-Fi has advantages over other systems, one drawback of this technology is its high-power consumption. It also takes some time to establish a connection, which may create problems, especially for blind persons [33].

The authors in [14] presented a better system for indoor navigation of blind persons. Initially designed for locating nurses and doctors or other patients in a hospital, it can also be used to find blind persons, as the technology is based on a Wi-Fi system. The system is comprised of two kinds of navigation or localization systems, one used for nurses or doctors and the other for patients. The position of patients is located on their call button, a transceiver module, a small USB stick equipped with TCM 310, and a computer, and is identified through the trilateration technique. The nurse’s navigation prototype, in contrast, is comprised of mobile phones, Wi-Fi, and alarms. The nurse’s position is established using the “time of arrival (TOA)” method. The results for position precision among patients and nurses came out to be 3 m and 2 m, respectively.

**Figure 3:**
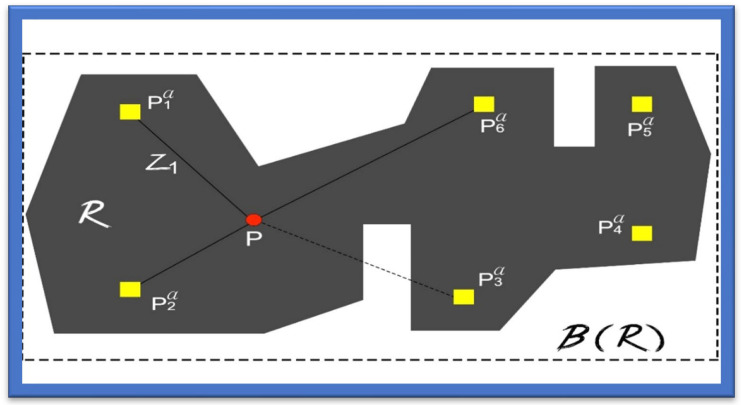
Navigation Using Wi-Fi Triangulation.

In [34], Wi-Fi triangulation was used to help blind people move about places near their location. In this article, the system was designed to assist in detecting and locating indoor areas so that no human or manual assistance is required to reach any location. The system mainly uses Wi-Fi signals. Wi-Fi was set up in various malls, buildings, and commercial places that had many strong Wi-Fi access Internet points to help the blind and the visually impaired navigate those indoor locations. The primary purpose of the proposed design was to identify the locations within the building and colleges, all of which was completed using the Wi-Fi triangulation approach. As the Wi-Fi is fixed within the buildings at each point, it becomes helpful for the signals to be transmitted from the Wi-Fi access points, which can be accessed via a smartphone or any other smart gadget helping to navigate as shown in Figure 3.

For further assistance in the localization of blind persons, a navigational map was created. With its help, a site is mentioned by detecting the Wi-Fi spot in a specified place which is further shown up by the point/dot that can be understood easily. Using this dot, a place can be navigated to efficiently. However, the important thing is to design an exact map of the place.

### 2.3. Obstacles Recognition Method

Many methods are used to detect obstacles. These methods can be explained as follows.

#### 2.3.1. The Technology of a Camera-Based Image Processing Navigation System

This real-time technology alerts the visually impaired to any dynamic or static obstacles within a range of meters; it works without smartphones and uses a camera to detect background motion. This system requires no previous knowledge of the obstacle size, position, or shape [35]. While the camera-based image processing system is a good choice, its drawbacks are its high processing power, which makes it costly and challenging to use. The camera takes real-time images, which are processed to detect obstacles within a range of some meters quickly. In [36], the authors explained how cutting-edge machine vision methodologies offer tools to assist with basic human needs such as psychological capacity, individual movement, and activities of daily living. They also discussed how data preprocessing, pattern classification, and computer vision work along with robotic systems to offer these methods. Users will gain knowledge of new computer vision methods for assisting mental abilities, strategies for assessing behaviour, and of the development of sophisticated rehabilitative solutions that mimic human movement and engagement using intelligent displays with interactive virtual technologies. The researcher’s goal was to examine the vision replacement perspective that focuses on image processing and computer vision [37]. This article’s main objective was to investigate gadgets that use detectors and codes. The report showed that a few assistive devices are sold commercially. The perceptions and financial benefits of blind people influence the most generally available types of technology.

Another mobile camera-based navigation system for the visually impaired was proposed in [38]. This system uses pre-stored images of the floor to guide people in finding the obstacle-free route by comparing current floor images with pre-stored images and checking for introduced obstacles. Table 2 provides detailed information on camera-based image processing navigation systems with proposed models.

#### 2.3.2. Approach of Ultrasonic Sensor-Based Navigation Systems for Blinds

Ultrasonic sensor-based technology is the last wireless category to be discussed within the scope of this paper. This technology is based on “ultrasound waves”. The distance between the ultrasonic transmitter and reflector is calculated based on the “arrival and reflected” time. The drawback of this technology is the “short communication range”, and the inclusion of L.O.S. among obstacles and sensors is considered essential [39].

In [38], a “smart environment explorer (SEE)”-based stick system was designed and proposed for blind people. The system guides the visually impaired and helps enhance the ability for “space consciousness”. In addition, the proposed system helps estimate the position of the blind person or of any user. It helps identify any hurdle in their way. The method comprises an ultrasonic sensor, accelerometer, camera, G.P.S., wheel encoder, smartphone, and routing protocol. It makes use of the “reduced inertial sensor system (R.I.S.S.)-G.P.S.-linear Kalman filter (L.K.F) algorithm” for tracking estimation. The initial results of the system have proved successful for blind people in walking, tracking, and detecting traffic lights, even for very short-range tracking. Table 3 incorporates the study of different algorithms used in wireless technology for the visually impaired.

#### 2.3.3. Technology of the Speech-to-Text-Based Navigation System

Using various computational devices, speech-to-text-based navigation systems are developed technologies that make blind or visually impaired persons recognize and translate the verbal/spoken language into text. The S.T. technology is also known as “automatic speech recognition (A.S.R.)” and “Computer Speech Recognition (C.S.R.)”.

#### 2.3.4. Technology of the Text-to-Speech-Based Navigation System

Text-to-Speech (TTS) technology [40] is the process where the electronic device is made to render the text from any document or image into speech. For this purpose, the natural language processing phenomenon is used. Several examples of text-reading applications include label reading, the BrickPi reader, voice stick, or pen reading. In addition to that, the finger-reading technique has also been developed under TTS technology. Table 4 provides initial information on audio assistance and speech recognition inputs and their costs and limitations.

### 2.4. Methods for Informing the Visually Impaired

Several assistive methods assist the visually impaired in conveying environmental information. The most common feedback is audio and vibration; the audio methods can be further classified into generic audio and bone conduction. The authors of [41] examined a variety of cellphone technologies targeting persons with visual impairments. According to their research, interest in developing and designing methods to assist and enhance persons who are VI is growing. They looked at several assistive technology options and considered this study to be a future attempt to handle various technology tools in a cohesive system that gives the necessary assistance for persons with VI, even though the tools are rarely integrated and are aimed at multiple programs. They advised creating a solid system that can coordinate a variety of programs that offer the best assistance and support possible for persons with VI, including those who are blind or have low vision.

#### 2.4.1. Haptic Feedback

A commercialized product called WeWalk helps the VI detect obstacles above the chest level by utilizing an ultra-sonic sensor [42]. This can also be connected to a cane using Bluetooth with the help of the mobile application. The communication with the user uses haptic feedback to make them aware of the surroundings. Another commercially available product, SmartCane [43], uses light and ultrasonic sensors to inform the VI user about the environment and uses audio and haptic feedback/vibration to warn the user of the surroundings. In [44], the author presented a technology to assist the VI person that uses haptic feedback. This unit detects obstacles above the knee via ultra-sonic sensors. In [45], the author presented another technology for VI users that uses haptic feedback/vibration to warn the VI person of the obstacle.

Haptic feedback can be divided into three categories: vibrotactile, shape-changing, and kinesthetic feedback.

##### Vibrotactile

This feedback can be used on various body parts such as the shoulder [46], heads [47], waists [48], feet [49], wrists [50], hands [51], etc., which is the most common type of haptic feedback. Direct and indirect mapping can define the relationship between direction and vibration. In indirect mapping, the researchers used a pattern of vibration that indicates the movement, such as in Pocket Navigator [52]; two short pulses indicate movement ahead. In mapping, a direct spatial relationship exists between the target direction and location of vibration [53].

##### Shape-Changing Feedback

The direction indication can also be carried out using a feedback method called shape-changing, such as the Tactile Handle. This device is based on the shape of a barbell with proximity sensors, actuators, and an embedded micro-controller. This controller is used to match the phalanxes of a finger. The direction is indicated directly by torsion and vibration [54].

##### Kinesthetic Feedback

The directional cues can also be provided by kinesthetic feedback. In [55], the author presented a new indicator for direction. The method of kinesthetic perception is based on the hand of haptic direction. This method is called a pseudo-attraction force which uses a nonlinear relationship, and a force sensation is generated between physical and perceived acceleration. Another [56] way was presented by the author based on the haptic feedback method in which kinesthetic stimuli are provided to help the VI user navigate.

#### 2.4.2. Generic Audio Feedback

In [57], the author presented an assistive aid called the Smart White Cane that uses audio and haptic feedback methods to communicate information to VI users about their surroundings. In this, the ultra-sonic sensors are used to detect the downfalls, potholes, pits, staircases both up and down, low knee-level obstacles and obstacles above the waste. In this, the feedback method of vibration and pre-recorded audio is used.

##### Systems with 3D Sound

In the system introduced by the author of [58] for the visually impaired, the user is helped by creating tactile and auditory representation methods that give information about the surroundings through haptic and audio feedback. This device is based on molded headgear and has a stereo camera and earphones.

##### Sonification Method

Another method used to convey information to the visually impaired is sonification, which is based on non-speech audio and transmits information to the user. It transforms data relations into perceived relations using an acoustic signal [59]. Sonification can be further divided as follows:

**i** **.**  **Preliminary Parameter Mapping Sonification**

This is referred to as a subcategory of sonification which maps the parameters of data into the parameters of sound. This method is widely used [60]. Interaction based on audio for E.T.A. can be considered a specific application for this type of sonification. Parameter Mapping Sonification is based on three elements: data parameters, sound parameters, and a function that is used to map these parameters.

**ii** **.**  **Image Sonification**

It is a difficult task to obtain information about distance by using depth images and to use this information to avoid obstacles. In image sonification, the depth images are converted directly into sound. The parameters of pixels are mapped into the sound in the image sonification.

**iii** **.**  **Obstacle Sonification**

In this method, the information about the obstacles is transmitted to the VI person. This method has less redundancy as compared to the raw depth images in which images are converted directly to the sound.

#### 2.4.3. Bone Conduction Feedback

In [61], the author used the bone conduction method, which is based on the configuration of an infrared sensor, a compass, an ultrasonic sensor, and a triaxial accelerometer, all of which are mounted on a white cane. The data are collected through the sensor and transmitted to the user’s smartphone, and the bone conduction headphones notify the user of the obstacles.

In [62], the author introduced another method based on the conduction feedback. The Smart Cane can detect the obstacles and faces of friends and family members within 10 m. A Bluetooth earpiece based on bone conduction is used to convey information about the obstacles.

## 3. Tools Based on Navigation Systems for the Visually Impaired

This literature examination has given an insight into the most advanced navigation systems for blind users, categorizing the methods according to the technology involved [63]. Their studies indicated several disadvantages connected to user involvement and adjustment to the new scheme. Additionally, they considered the issues of comfort, confidence, mobility, and adaptive multi-feedback possibilities for a VI person. Several factors may have contributed to the lack of acceptance of the technology by the blind and visually impaired population and the resistance of the intended users to using them. Due to the ophthalmic-centric nature of the organizations, individuals with vision impairments typically encounter some challenges when viewing museums. The situation is made worse by the frequent inaccessibility of exhibits and replicas from a physical, intellectual, and sensory perspective. This is made worse by the inability to use information for communication technology and or local or global resources for substitute or intensifier communication that can enable different interactions for sighted visitors. The research examines assistive technology applications for the design of multimodal exhibits and links them to visitor experiences. Based on a survey of the literature, this article [64] intends to advance the topic of mobility in museums by presenting an overview of the perceptions and needs of blind and visually impaired visitors to museums.

### 3.1. Braille Signs Tools

Individuals with visual impairments must remember directions, as it is difficult to note them down. If the visually impaired lose their way, the main way forward is to find somebody who can help them. While Braille signs can be a decent solution here, the difficulty with this methodology is that it cannot be used as a routing tool [65]. These days, numerous public regions, such as emergency clinics, railroad stations, instructive structures, entryways, lifts, and other aspects of the system, are outfitted with Braille signs to simplify the route for visually impaired individuals. Regardless of how Braille characters can help visually impaired individuals know their area, they do not help to find a path.

### 3.2. Smart Cane Tools

**Figure 4:**
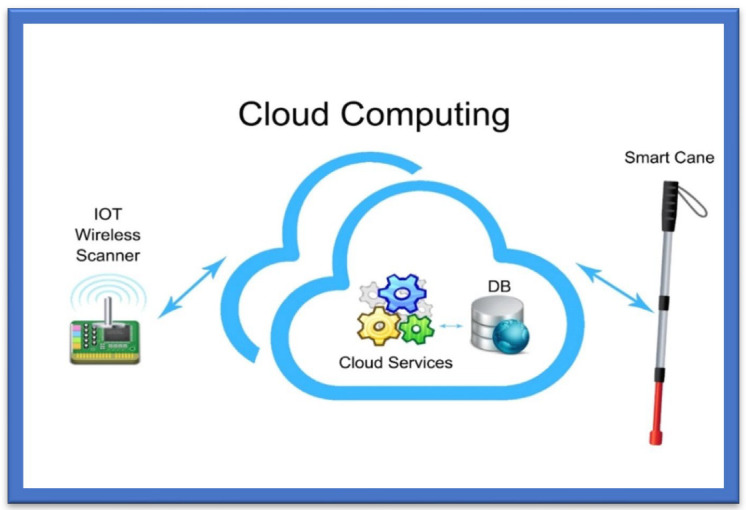
Smart Cane Navigation System.

Smart canes help the visually impaired navigate their surroundings and detect what appears in front of them, either big or small, which is impossible to detect and identify (the size) with simple walking sticks. A smart guiding cane detects the obstacle, and the microphone produces a sound in the intelligent system deployed in the cane. The cane also helps to detect a dark or bright environment [66]. For indoor usage, an innovative cane navigation system was proposed [67] that uses IoT and cloud networks. The intelligent cane navigation system is capable of collecting the data transmitted to the cloud network; an IoT scanner is also attached to the cloud network. The concept is shown in Figure 4.

The Smart Cane Navigation System comprises the camera, microcontrollers, and accelerometers that send audio messages. A cloud service is exploited in the navigation system to assist the user in navigating from one point to another. This navigation system fundamentally assists visually impaired or blind people in navigating and detecting the fastest route. Nearby objects are detected, and users are warned via a sound buzzer and a sonar [63]. Cloud services acquire the position of the cane and route to the destination, and these data go from the Wi-Fi Arduino board to the cloud. The system then uses a Gaussian model for the triangular-based position estimation. The cloud service is linked to the database that stores the shortest, safest, and longest paths. It outputs three lights: red, when objects are greater than 15 m; yellow, between 5 and 15 m; and green, less than 15 m. Distance is calculated by sound emission and echoes, which is the cheapest way of calculating distance, and a text-to-audio converter warns of possible hurdles or obstacles. Experiments have shown that this navigation system is quite effective in suggesting the fastest/shortest route to the users and identifying the hurdles or obstacles:

- The system uses a cloud-based approach to navigate different routes. A Wi-Fi Arduino board in this cane connects to a cloud-based system;
- The sound echoes and emissions are used to calculate the distance, and the user obtains a voice-form output;
- The system was seen to be very efficient in detecting hurdles and suggesting the shortest and fastest routes to the visually impaired via a cloud-based approach.

### 3.3. Voice-Operated Tools

This outdoor voice-operated navigation system is based on G.P.S., ultrasonic sensors, and voice. This outdoor navigation system provides alerts for the current position of the users and guidance for traveling. The problem with this system is that it failed in obstacle detection and warning alerts [68]. Another navigation system uses a microcontroller to detect the obstacles and a feedback system that alerts the users about obstacles through voice and vibration [69].

### 3.4. Roshni

Roshni is an indoor navigation system that navigates through voice messages by pressing keys on a mobile unit. The position of the users in Roshni is identified by sonar technology by mounting ultrasonic modules at regular intervals on the ceiling. Roshni is portable, free to move anywhere, and unaffected by environmental changes. It needs a detailed interior map of the building that limits it only to indoor navigation [66].

Roshni application tools are easy to use, as the system operates by pressing mobile keys and guides the visually impaired using voice messages. Since it remains unaffected by a change in environment, it is easily transportable. The system is limited to indoor locations, and the user must provide a map of the building before the system can be used.

### 3.5. RFID-Based Map-Reading Tools

RFID is the fourth category of wireless technology used to facilitate visually impaired persons for indoor and outdoor activities. This technology is based on the “Internet of Things paradigm” through an IoT physical layer that helps the visually impaired navigate in their surroundings by deploying low-cost, energy-efficient sensors. The short communication range leaves this RFID technology incapable of being deployed in the landscape spatial range. In [70], an indoor navigation system for blind and older adults was proposed, based on the RFID technique, to assist disabled people by offering and enabling self-navigation in indoor surroundings. The goal of creating this approach was to handle and manage interior navigation challenges while taking into consideration the accuracy and dynamics of various environments. The system was composed of two modules for navigation and localization—that is, a server and a wearable module containing a microcontroller, ultrasonic sensor, RFID, Wi-Fi module, and voice control module. The results showed 99% accuracy in experiments. The time the system takes to locate the obstacle is 0.6 s.

Another map-reading system based on RFID provides solutions for visually disabled persons to pass through public places using an RFID tag grid, a Bluetooth interface, a RFID cane reader, and a personal digital assistant [71]. This system is costly, however, and there is a chance of collision in heavy traffic. A map-reading system is relatively expensive because of the hardware units it includes, and its limitation is that it is unreliable for areas with heavy traffic.

Another navigation system based on passive RFID proposed in [72] is equipped with a digital compass to assist the visually impaired. The RFID transponders are mounted on the floor, as tactile paving, to build RFID networks. Localization and positioning are equipped with a digital compass, and the guiding directions are given using voice commands. Table 5 incorporates detailed information about RFID-based navigation tools with recommended models for the visually impaired.

### 3.6. Wireless Network-Based Tools

Wireless network-based solutions for navigation and indoor positioning include various approaches, such as cellular communication networks, Wi-Fi networks, ultra-wideband (U.W.B.) sensors, and Bluetooth [73]. The indoor positioning is highly reliable in the wireless network approach and easy to use for blind persons. Table 6 summarises various studies based on the wireless networks for VI people.

### 3.7. Augmented White Cane Tools

**Figure 5:**
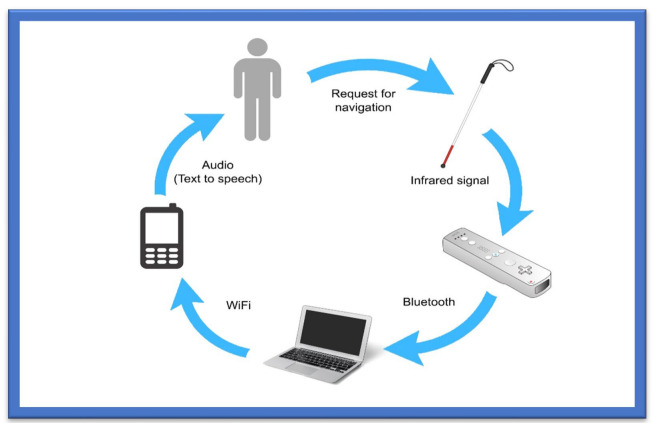
Augmented White Cane.

The augmented white cane-based system is an indoor navigation system specifically designed to help the visual impaired move freely in indoor environments [74]. The prime purpose of the white cane navigation system is to provide real-time navigation information, which helps the users to make decisions appropriately, for example on the route to be followed in an indoor space. The system obtains access to the physical environment, called a micro-navigation system, to provide such information. Possible obstacles should be detected, and the intentional movements of the users should be known to help users decide on movements. The solution uses the interaction among several components. The main components comprising this system are the two infrared cameras. The computer has a software application, in running form, which coordinates the system. A smartphone is needed to deliver the information related to navigation, as shown in Figure 5.

The white cane helps determine the user’s position and movement. It includes several infrared L.E.D.s with a button to activate and deactivate the system. The cane is the most suitable object to represent the position, assisted by an Augmented Objects Development Process (AODeP). To make an object augmented, many requirements can be identified: (1) the object should be able to emit the infrared light that the Wiimote could capture, (2) the user should wear it to obtain his location or position, (3) it should be smaller in size so that it does not hinder the user’s movement, and (4) it should minimize the cognitive effort required to use it:

- The white cane provides real-time navigation by studying the physical indoor environment by using a micro-navigation system;
- The two infrared cameras and a software application make indoor navigation more reliable and accurate;
- The whole system takes input through infrared signals to provide proper navigation.

### 3.8. Ultrasonic Sensor-Based Tools

This ultrasonic sensor-based system comprises a microcontroller with synthetic speech output and a portable device that guides users to walking points. The principle of reflection of a high frequency is used in this system to detect obstacles. These instructions or guidelines are given in vibro-tactile form for reducing navigation difficulties. The limit of such a system is the blockage of the ultrasound signals by the wall, thus resulting in less accurate navigation. A user’s movement is constantly tracked by an RFID unit using an indoor navigation system designed for the visually impaired. The user is given the guidelines and instructions via a tactile compass and wireless connection [75].

### 3.9. Blind Audio Guidance Tools

**Figure 6:**
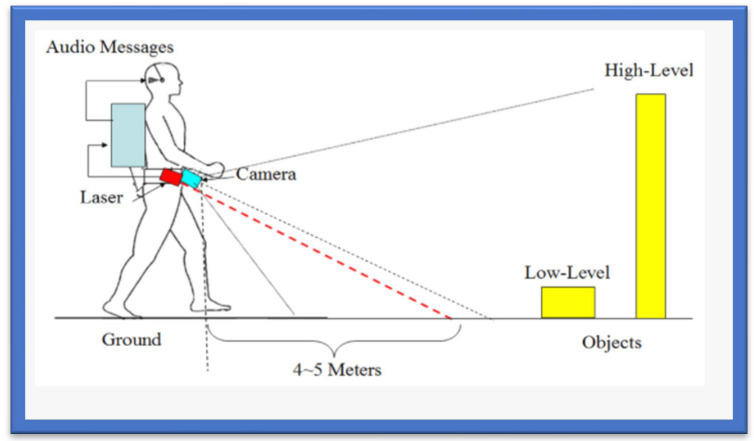
The Blind Audio Guidance System.

The blind audio guidance system is based on an embedded system, which uses an ultrasonic sensor for measuring the distance, an A.V.R sound system for the audio instructions, and an I.R.R. sensor to detect objects as shown in Figure 6. The primary functions performed by this system are detecting paths and recognizing the environment. Initially, the ultrasonic sensors receive the visual signals and then convert them into auditory information. This system reduces the training time required to use the white cane. However, the issue concerns identifying the users’ location globally [76]. Additionally, Table 7 provides various blind audio guidance system features relating to Distance Measurement, Audio Instructions, and Hardware Costs.

### 3.10. Voice and Vibration Tools

This system is developed using an ultrasonic sensor for the detection of obstacles. People with any visual impairment or blindness are more sensitive to hearing than others, so this navigation system gives alerts via voice and vibration feedback. The system works both outdoors and indoors. The alert mobility of the users and different intensity levels are provided [77]. Table 8 incorporates the properties of voice and vibration navigation tools used for the visually impaired.

### 3.11. RGB-D Sensor-Based Tools

**Figure 7:**
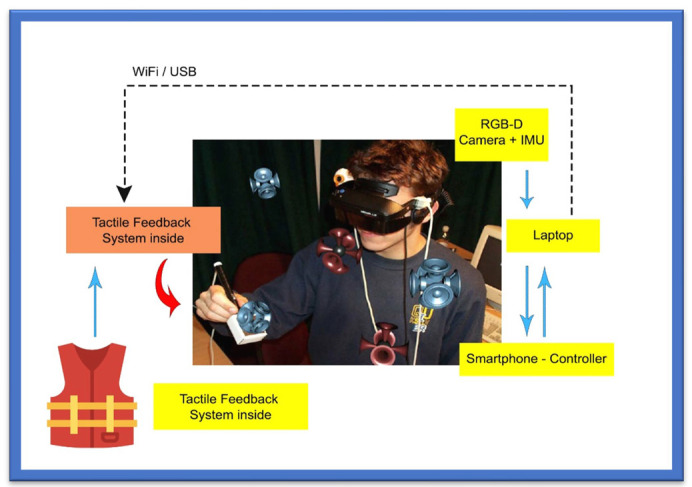
RGB-D Sensor-Based System.

This navigation system is based upon an RGB-D sensor with range expansion. A consumer RGB-D camera supports range-based floor segmentation to obtain information about the range as shown in Figure 7. The RGB sensor also supports colour sensing and object detection. The user interface is given using sound map information and audio guidelines or instructions [78,79]. Table 9 provides information on RGB-D sensor-based navigation tools with their different properties for VI people.

### 3.12. Cellular Network-Based Tools

A cellular network system allows mobile phones to communicate with others [80]. According to a research study [81], a simple way to localize cellular devices is to use the Cell-ID, which operates in most cellular networks. Studies [82,83] have proposed a hybrid approach that uses a combination of wireless local area networks, Bluetooth, and a cellular communication network to improve indoor navigation and positioning performance. However, such positioning is unstable and has a significant navigation error due to cellular towers and radiofrequency signal range. Table 10 summarizes the information based on different cellular approaches for indoor environments with positioning factors.

### 3.13. Bluetooth-Based Tools

Bluetooth is a commonly used wireless protocol based on the IEEE 802.15.1 standard. The precision of this method is determined by the number of connected Bluetooth cells [84]. A 3D indoor navigation system proposed by Cruz and Ramos [85] is based on Bluetooth. In this navigation system, pre-installed transmitters are not considered helpful for applications with critical requirements. An approach that combines Bluetooth beacons and Google Tango was proposed in [86]. Table 11 provides an overview of Bluetooth-based approaches used for the visually impaired in terms of cost and environment.

### 3.14. System for Converting Images into Sound

Depth sensors generate images that humans usually acquire with their eyes and hands. Different designs convert spatial data into sound, as sound can precisely guide the users. Many approaches in this domain are inspired by auditory substitution devices that encode visual scenes from the video camera and generate sounds as an acoustic representation known as “soundscape”. Rehri et al. [87] proposed a system that improves navigation without vision. It is a personal guidance system based on the clear advantage of virtual sound guidance over spatial language. The authors argued that it is easy and quick to perceive and understand spatial information.

In Nair et al. [88], a method of image recognition was presented for blind individuals with the help of sound in a simple yet powerful approach that can help blind persons see the world with their ears. Nevertheless, image recognition using the sound process becomes problematic when the complexity of the image increases. At first, the sound is removed using Gaussian blur. In the second step, the edges of images are filtered out by finding the gradients. In the third step, non-maximum suppression is applied to trace along the image edges. After that, threshold values are marked using the canny edge detector. After acquiring complete edge information, the sound is generated.

These different technologies are very effective for blind and the visually impaired and help them feel more confident and self-dependent. They can move, travel, play, and read books more than sighted people do. Technology is growing and is enhancing the ways B.V.I. communicates to the world more confidently.

### 3.15. Infrared L.E.D.-Based Tools

Next comes the infrared L.E.D. category, suitable for producing periodic signals in indoor environments. The only drawback of this technology is that the “line of sight (L.O.S.)” must be accessible among L.E.D. and detectors. Moreover, it is a technology for short-range communication [89]. In this study, a “mid-range portable positioning system” was designed using L.E.D. for the visually impaired. It helps determine orientation, location, and distance to destination for those with weak eyesight and is 100% accurate for the partially blind. The system comprises two techniques: infrared intensity and ultrasound “time of flight T.O.F.”. The ultrasonic T.O.F. structure comprises an ultrasonic transducer, beacon, and infrared L.E.D. circuits.

On the other hand, the receiver includes an ultrasonic sensor, infrared sensor, geomagnetic sensor, and signal processing unit. The prototype also includes beacons of infrared L.E.D. and receivers. The system results showed 90% accuracy for the fully visually impaired in indoor and outdoor environments.

#### 3.15.1. Text-to-Speech Tools

**Figure 8:**
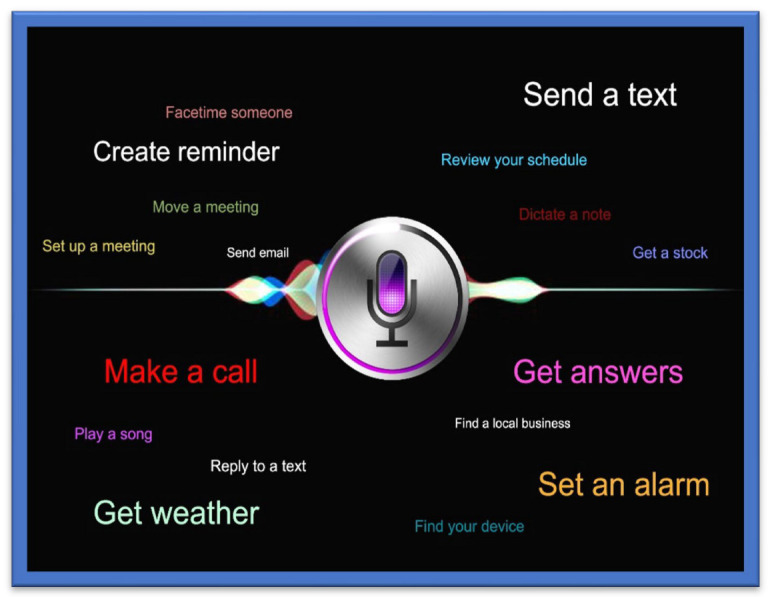
Siri for iPhone.

One of the most used and recently developed Text-to-Speech Tools (TTS) is the Google TTS, a screen reader application that Google has designed and that uses Android O.S.S. to read the text out loud over the screen. It supports various languages. This device was built entirely based upon “DeepMind’s speech” synthesis expertise. In this tool, the API sends the audio or voice to the person in almost human voice quality (Cloud Text-to-Speech, n.d). OpenCV tools and libraries have been used to capture the image, from which the text is decoded, and the character recognition processes are then completed. The written text is encoded through the machine using O.C.R. technology. The OpenCV library was recommended for its convenience of handling and use compared to the other P.C.C. or electronic devices platforms [90]. An ultrasonic sensor-based TTS tool was designed to give vibration sensing for the blind to help them to easily recognize and identify a bus name and its number at a bus stop using audio processing techniques. The system was designed using M.A.T.L.A.B. for implementing image capture. Most simulations are performed using O.C.R. in M.A.T.L.A.B. to convert the text into speech [91]. A text-to-voice technique is also presented; most of the commercial G.P.S. devices developed by Inc., such as TomTom Inc., Garmin Inc., etc., use this technique. Real-time performance is achieved based on the spoken navigation instructions [92]. An ideal illustration of combining text-to-voice techniques and voice search methods is Siri, which is shown in Figure 8 Siri is available for iOS, an operating system (O.S.S.) for Apple’s iPhone. It is easy to interact or talk to Siri and receive a response in a human-like voice. This system helps people with low vision and people who are blind or visually impaired to use it in daily life with both voice input and synthesized voice output.

A Human–Computer Interface (HCI)-based wearable indoor navigation system is presented by [93]. An excellent example of an audio-based system is Google Voice search. To effectively use such systems, proper training is required [26,94].

#### 3.15.2. Speech-to-Text Tools

Amazon has designed and developed an S.T.T. tool named “Amazon transcribe” that uses the deep learning algorithm known as A.S.R., which converts the voice into text in a matter of seconds and does so precisely. These tools are used by the blind and the visually impaired, and they are also used to translate customer service calls, automate subtitles, and create metadata for media assets to generate the searchable archive [15]. I.B.M. Watson has also developed its own S.T.T. tool to convert audio and voice to text form. The developed technology uses the DL AI algorithm, which applies the language structure, grammar, and composition of voice and audio signals to transcribe and convert the human voice/audio into written text [95].

Based on multiple tools and techniques incorporated in assistive technologies for visually impaired users, we have identified what is lacking in the current systems. Table 12 shows the complete evaluation and analysis of current systems to help visually impaired users confidently move in their environment.

### 3.16. Feedback Tools for VI

In this section, different feedback tools for VI people are explained.

#### 3.16.1. Tactical Compass

The feedback for effective and accurate direction in an electronic travel aid for VI is a challenging task. The authors of [96] presented a Tactile Compass to guide the VI person during the traveling to address this problem. This compass is a handheld device that guides a VI person continuously by providing directions with the help of a pointing needle. Two lab experiments that tested the system demonstrated that a user can reach the goal with an average deviation of 3.03°.

#### 3.16.2. SRFI Tool

To overcome the information acquisition problem in VI people, the authors of [97] presented a method based on auditory feedback and a triboelectric nanogenerator. This tool is called a ripple-inspired fingertip interactor (S.R.F.I.) and is self-powered. It assists the VI person by giving feedback to deliver information, and due to its refined structure, it gives high-quality text information to the user. Based on three channels, it can recognise Braille letters and deliver feedback to VI about information acquisition.

#### 3.16.3. Robotic System Based on Haptic Feedback

To support VI people in sports, the authors of [98] introduced a robotic system based on haptic feedback. The runner’s position is determined with the help of a drone, and information is delivered with the help of the left lower leg haptic feedback, which guides the user in the desired direction. The system is assessed outdoors to give proper haptic feedback and is tested on three modalities: vibration during the swing, stance, and continuous.

#### 3.16.4. Audio Feedback-Based Voice-Activated Portable Braille

A portable device named Voice Activated Braille helps give a VI person information about specific characters. Arduino helps direct the VI person. This system can be beneficial for VI by guiding them. It is a partial assistant and helps the VI to read easily.

#### 3.16.5. Adaptive Auditory Feedback System

This system helps a VI person while using the desktop, based on continuous switching between speech and the non-speech feedback. Using this system, a VI person does not need continuous instructions. The results of sixteen experiments that assessed the system revealed that it delivers an efficient performance.

#### 3.16.6. Olfactory and Haptic for VI person

The authors of [99] introduced a method based on Olfactory and Haptic for VI people. This method is introduced to help VI in entertainment in education and is designed to offer opportunities to VI for learning and teaching. A 3D system, it can be used to touch a 3D object. Moreover, the smell and sound are released from the olfactory device. This system was assessed by the VI and blind people with the help of a questionnaire.

#### 3.16.7. Hybrid Method for VI

This system guides the VI person in indoor and outdoor environments. A hybrid system based on the sensor and traditional stick, it guides the user with the help of a sensor and auditory feedback.

#### 3.16.8. Radar-Based Handheld Device for VI

A handheld device based on radar has been presented for VI people. In this method, the distance received by the radar sensor is converted to tactile-based information, which is mapped into the array based on the vibration actuators. With the help of an information sensor and tactile stimulus, the VI user can be guided around obstacles. Table 12 gives a detailed overview of the navigation application for the visually impaired. The study involves various models and applications with their feasibility and characteristics report and discusses the merits and drawbacks of every application along with the features.

**Table 12:** Navigation Applications for Visually Impaired Users.

Ref. Paper	Name	Components/Application	Device/Application Features	Price/Usability and Wearability/User Feedback	Drawbacks/User Acceptance	Specific Characteristics
[100]	Maptic	Sensor device, user-friendly feedback capabilities, cell phone	(1) Obstacle detection for upper body Instant navigation guidance	Unknown/Wearable/Instant feedback	Lower body and ground obstacles detection	Object detection
[101,102]	Microsoft Soundscape	Cell phone, beacons application	(1) Instant navigation guidance Follows preferences	Free/Handheld device/Audio	No obstacle detection	Ease of use
[103]	SmartCane	Sensor, SmartCane, vibration function	Detects obstacles	Affordable/Handheld device/Quick feedback	Navigation guidance not supported	Detection speed Accuracy of obstacle detection
[104]	WeWalk	Sensor device, cane feedback, smartphone	(1) Detects obstacles (2) Navigation (3) Follows preference. Use of public transportation	Price starts from USD 599/Wearable (weighs about 252 g/0.55 pounds Audio-related and instant feedback	No obstacle detection and number of scenario description when in use	Ease of use Prioritization of user’s requirements Availability of application
[105]	Horus	Headphone with the bone-conducted facility Supports two cameras with an additional battery and powerful GPU.	(1) Detects obstacles (2) Face recognition and reads text (3) Scene detection and description	Price USD 2000/Wearable/Audio related feedback	Navigation guidance not supported	Less power consumption Reliability and voice assistant Accuracy of obstacles
[106]	Ray Electronic Mobility Aid	Ultrasonic device	(1) Detect Obstacles	Price USD 395/Handheld weighs 60 g/Audio-related and instant feedback	Navigation guidance not supported	Object classification accuracy Incorporated user feedback Accurate obstacle detection and classification
[107]	UltraCane	Dual Range, Ultrasonic with narrow beam, Cane	(1) Detect Obstacles	Price USD 590/Handheld/instant feedback	Navigation guidance not supported	Power consumption Battery life Ultrasonic Cane
[108]	BlindSquare	Cell Phone	(1) Navigation (2) Follows the interest preference Use the Public transportation	Price USD 39.99/Handheld/Audio feedback enabled	Navigation guidance not supported	Ease of use Availability of application
[109]	Envision Glasses	Wearable glasses with additional cameras	(1) Text read (2) Description of scenes (3) Scan Barcodes, Color detections, Help in finding belongings Facial detection helps to make calls, voice commands to share text	Price USD 2099/wearable device weighs 46 g/Audio oriented	No Obstacle detection Navigation guidance not supported	Object classification Bar code scanning Voice supported application Performance
[110]	Eye See	Helmet with integrated cameras and laser	(1) Read text (2) Obstacle detection Description of people	Unknown/wearable/Audio	Navigation guidance not supported.	Obstacle detection, O.C.R. Incorporated, Text to voice conversion.
[111]	Nearby Explorer	Cell phone	(1) Navigation (2) Interest preferences support (3) Tracking Objects identification	Free/Handheld/Audio and instant feedback	No obstacle detection	Object identification User’s requirement prioritization Tracking history
[112]	Seeing Eye G.P.S	Cell phone	(1) Navigation (2) Interest preferences support	Unknown/Handheld/Audio	No obstacle detection	User preference G.P.S. based navigation
[113]	PathVu Navigation	Cell phone	Give alerts about sidewalk problem	Free/Handheld/Audio	No obstacle detection Navigation guidance not supported	Catering offside obstacles through alerts
[114]	Step-hear	Cell phone	(1) Navigation (2) Uses public transport	Free/Handheld/Audio	No obstacle detection	Availability of application to public G.P.S. based navigation
[115]	InterSection Explorer	Cell phone	Streets information	Free/Handheld/Audio	No obstacle detection Navigation guidance not supported	Predefined routes.
[116]	LAZARILLO APP	Cell phone	(1) Navigation (2) Uses public transport (3) Interest preferences support	Free/Handheld/Audio	No obstacle detection	Availability of application to public G.P.S. based navigation User requirement prioritization
[117]	Lazzus APP	Cell phone	(1) Navigation (2) Interest preferences support Crossing information	Price USD 29.99/Handheld/Audio	No obstacle detection	User requirement preference Predefined crossings on routes.
[118]	Sunu Band	Sensor’s device	Upper detection of body	Price USD 299/Handheld/Instant feedback	Lower body and ground obstacles are not detected	Object detection
[119]	Ariadne G.P.S.	Cell phone	(1) Navigation Map explorer	Price USD 4.99/Handheld/Audio	No obstacle detection	Accurate Map Exploration G.P.S. based navigation
[120]	Aira	Cell phone	Sighted person support	Price USD 99 for 120 min/ Handheld/Audio	Expensive to use and privacy concerns	Tightly coupled for security Ease of use
[121]	Be My Eyes	Cell Phone	Sighted person support	Free/Handheld/Audio	Privacy concerns	Ease of use
[122]	BrainPort	The handheld video camera controller	Detection of objects	Expensive/wearable and handheld/Instant Feedback	No navigation guidance	Accurate detection of objects/obstacles

## 4. Discussion

Several research studies were conducted on various navigational systems that have been created over time to assist the visually impaired and blind, but of which only a few remain in use. Although most of the methods make sense in theory, in practice they may be excessively complicated or laborious for the user. This evaluation analysis has been divided into sections according to specific characteristics, including recording methods, smart response to objects, physical hardware, transmission range, detection limit, size, and cost efficiency. These preselected standards described in this study are chosen because they assess system performance. Navigating through various situations is difficult for those with vision impairments, who must be aware of the objects and landscape in the immediate environment, such as people, tables, and dividers. Likewise, the inability to deal with such circumstances itself adversely affects the sense of freedom of the visually impaired, who have little opportunity to find their way in a new environment.

A guide is always needed for the outdoor environment. However, it is not a good solution due to dependency on others. One can request directions for a limited time at some place but asking people for direction every time causes difficulties to move freely. Numerous tools have been developed and effectively deployed to help impaired individuals avoid obstacles outdoors, such as intelligent canes and seeing-eye dogs, Braille signs, and G.P.S. systems. One approach to navigation systems is the adoption of neural networks. To help the visually impaired to move around, researchers have presented two deep Convolutional Neural Network models. However, this approach is not very efficient in terms of time complexity [90].

Some of the approaches for navigation systems have adopted sensory substitution methods, such as one based on LIDARs [87] or a vibrotactile stimulation [88] applied to the palms of the hand to direct users through a temporal sequence of stimuli. A vibrating belt with time-of-flight distance sensors and cameras was used to acquire better navigation [109]. An outdoor navigation system based on vision positioning to direct blind people was proposed in [115]. Image processing is used for identifying the path and obstacles in the path. An assistive-guide robot called an eye dog has been designed as an alternative to guide dogs for blind people [116].

In [90], the author designed an intelligent blind stick outfitted with an ultrasonic sensor and piezo buzzer that sounds an alarm when the user approaches an obstacle. A fusion of depth and vision sensors was proposed in [26], by which obstacles are detected using corner detection. At the same time, the corresponding distance is calculated using input received from the depth sensor. Further, the main problems for the visually impaired and blind are in finding their way in unfamiliar environments. While they strive to orient themselves in places where they had previously been, places such as shopping malls, train stations, and airports change almost daily. Blind people must orient themselves in a vast place full of distractions such as background music, announcement, various kinds of scents and moving people, etc. They cannot rely totally on their senses in such a place, and they cannot easily find help at any time, for example, by finding a clothing store on the fifth floor.

Although assistive technologies allow the visually impaired to navigate their surroundings freely and confidently, a significant concern that is usually neglected is the power consumption and charging time of such devices. Preferably, images of paths and pedestrians can be stored on mobiles to overcome these problems. Doing so will allow visually impaired users to identify the obstacles and routes automatically, thus consuming less power for capturing and processing. However, this solution takes up memory; shifting from mobile devices to the cloud, therefore, will ease this issue of memory consumption and sharing of updated data between devices [117]. Moreover, the fully charged device should last significantly more than 48 h to prevent difficulties with daily charging. Proper training should also be incorporated into the orientation and mobility of the visually impaired, as programs scheduled to train the blind and visually impaired to travel safely help in daily routine tasks and to fit into their community. Considering our previous work on serious games, we think that they may help in the training [118,119,120].

The training sessions helped the students to understand and learn different techniques for street crossings and public transportation with the help of a cane. Visually impaired students who are comfortable with having dog assistance are trained with the help of the application process. This training program is usually applied one-on-one in the student community, home, school, and workplace.

Most instructions and tasks are completed in group discussions and class settings. When organizing the plan for orientation and mobility assistive technologies for the visually impaired, the focus on training should not be neglected.

Some major categories of assistive technologies are:

(1) Public transportation crossing assistive technology, which allows the visually impaired to learn the door-to-door bus service or the public bus transport systems in their area. Training is required to understand assistive maps to reach the bus transport or understand routes according to the needs of the visually impaired user;

(2) Another assistive technology is the Sighted Guide, which allows visually impaired users to practice the skills required for traveling with the aid of a sighted person and gain the ability to train any sighted person to guide them safely through any environment;

(3) Similarly, Safe Travel assistive technology allows visually impaired users to travel safely in an unfamiliar environment. These assistive technologies help in detecting obstacles;

(4) Orientation-based assistive technology allows visually impaired users to become familiar with both indoor and outdoor environments with the help of an experienced instructor. Haptic and auditory feedbacks may help in guiding users [121,122,123].

(5) Cane Skills assistive technology allows visually impaired users to become familiar with canes and to identify objects without restricting their mobility. Orientation and mobility training for assistive technologies are therefore essential for visually impaired users because, without mobility, a user becomes homebound. With necessary training sessions, a visually impaired user learns the required skills and confidently navigates indoors and outdoors.

The evaluation gives a set of essential guidelines for detailing technological devices and the characteristics that must be incorporated into the methods to improve effectiveness. These criteria are described as follows:

- Basic: a method can be used relatively quickly without additional equipment assistance;
- Minimal cost: an affordable model must be built. Consequently, this design will be inaccessible to most individuals;
- Compactness: a compact size allows the device to be used by those with limited mobility;
- Reliable: the hardware and software requirements for the gadget must be compliant;
- Covering region: This gadget must meet the wireless needs of the individual both indoors as well as outside.

## 5. Conclusions and Future Work

Several of the latest assistive technologies for the visually impaired in the fields of computer vision, integrated devices, and mobile platforms have been presented in this paper. Even though many techniques under examination have been in relatively initial phases, most are being incorporated into daily life using state-of technology (i.e., smart devices). The proposed device aims to create an audio input and vibrations in the vicinity of obstructions in outdoor and indoor areas.

Our research review has comprehensively studied visually impaired users’ indoor and outdoor assistive navigation methods and has provided an in-depth analysis of multiple tools and techniques used as assistive measures for visually impaired users. It has also covered a detailed investigation of former research and reviews presented in the same domain and rendered highly accessible data for other researchers to evaluate the scope of former studies conducted in the same field. We have investigated multiple algorithms, datasets, and limitations of the former studies. In addition to the state-of-the-art tools and techniques, we have also provided application-based and subjective evaluations of those techniques that allow visually impaired users to navigate confidently indoors and outdoors.

In summary, detailed work is required to provide a reliable and comprehensive technology based on a Machine Learning algorithm. We have also emphasized knowledge transfer from one domain to another, such as driver assistance, automated cars, cane skills, and robot navigation. We also have insisted on training in assistive technology for visually impaired users. We have categorized assistive technologies and individually identified how training in each category could make a difference. However, previous work has ignored other factors involved in assistive technology, such as power consumption, feedback, and wearability. A technology that requires batteries and fast processing also require power consumption solutions relative to the device type. The researcher must determine the feasibility of running real-time obstacle detection through wearable camera devices. Various methods in the literature are “lab-based”, focusing on achieving accurate results rather than dealing with issues of power consumption and device deployment. Therefore, technologies that consume less power and are easy to wear in the future must be incorporated. These techniques should also be efficient enough to allow user to navigate effectively and confidently.

## Figures and Tables

**Table 1:** Comparison of Existing Technologies Based on Different Technologies.

Technology	Technique	Indoor	Outdoor	Requirement of Infrastructure
Direct Sensing	RFID	✓	✓	✓
	Bluetooth	✓	✓	✓
	I.R.R.	✓	-	✓
	Barcodes	✓	✓	✓
Dead Reckoning	IMU	✓	✓	-
Triangulation	From direct sensing	✓	✓	✓
	GNSS	-	✓	-
	Fingerprint	✓	✓	-
Pattern Recognition	Markers	✓	✓	-
	Natural Elements	✓	✓	-
	3D Sensing	✓	✓	-

**Table 2:** Summary of Various Camera-Based Navigation Technologies.

Ref. Paper	Title	Proposed Model
[27]	RGB-D camera-based navigation for the visually impaired	This system considers local and global frame localization and uses a feedback vest to deliver the navigational cues.
[28]	Visual odometry and mapping for autonomous flight using an RGB-D camera	This model is comprised of an rgb-d camera that provides a 3D point cloud. i.m.y. offers an initial orientation where the point cloud is downsampled to a 3D voxel grid display to the global frame using an odometry algorithm.
[29]	Enabling Independent Navigation for the visually impaired through a Wearable Vision-Based Feedback System	This model consists of a feedback module, a camera, and an embedded computer. It identifies a safe motion trajectory and communicates this safer route information with the user through a sequence of vibrations.

**Table 3:** Summary of Various Navigation Techniques.

Ref. Paper	Objective	Wireless Technology	Algorithm
[20]	Navigation	Bluetooth	Distributed Indoor Navigation
[31]	Navigation	RFID	User positioning and Tags
[23]	Route planning and navigation	Wi-Fi	Wi-Fi-based positioning
[30]	Navigation	Infrared L.E.D.	Obstacle detection
[32]	Obstacle detection and navigation	Ultrasonic sensor	RISS-GPS-LKF

**Table 4:** Summary of Various Audio-Assisted and Speech Recognition-Based Navigation Technologies.

Audio Assistance and Speech Recognition	
Voice Input	The systems such as Google Assistant and Siri take your voice as input to provide a precise output
Cost-Efficient	The visually impaired person only needs a smartphone installed with Speech Recognition assistance that will guide him in a human-like voice
Limitation	Speech Recognition and Assistance needs a stable wireless connection to provide you with voice output.

**Table 5:** Summary of Various RFID-Based Navigation Tools.

Ref. Paper	Title	Proposed Model
[41]	Mobile audio navigation interfaces for the blind	Drishti system combines the ultrasonic sensor for indoor navigation and G.P.S. for outdoor navigation for blind people.
[42]	BLI-NAV embedded navigation system for blind people	Comprised of G.P.S. and path detectors, which detect the path and determine the shortest obstacle-free route.
[43]	A pocket-PC-based navigational aid for blind individuals	Pocket PC-based Electronic Travel Aid (E.T.A.) warns users of obstacles via audio-based instructions.
[44]	A blind navigation system using RFID for indoor environments	A wireless mesh-based navigation system that warns the users about obstacles via a headset with microphone.
[45]	Design and development of navigation system using RFID technology	This system uses an RFID reader mounted on the end of the stick that reads the transponder tags installed on the tactile paving.

**Table 6:** Summary of Various Wireless-Network-Based Navigation Tools.

Ref. Paper	Model Used	Detailed Technology Used
[47]	Syndrome-Enabled Unsupervised Learning for Neural Network-Based Polar Decoder and Jointly Optimized Blind Equalizer	1. This neural network-based approach uses a polar decoder to aid CRC-enabled syndrome loss, B.P.P. decoder. 2. This approach has not been evaluated under varying channels.
[48]	Wireless Sensor Network Based Personnel Positioning Scheme used in Coal Mines with Blind Areas	This model determines the 3D positions by coordinate transformation and corrects the localization errors using real-time personal local with the help of a location engine.
[49]	A model based on ultrasonic spectacles and waist belts for blind people	This model detects obstacles within 500 cm. It uses a microcontroller-based embedded system to process real-time data gathered using ultrasonic sensors.

**Table 7:** Properties of the Blind Guidance System.

Blind Audio Guidance System	
Distance Measurement	The ultrasonic sensor facilitates the visually impaired by measuring distances accurately.
Audio Instructions	I.R.R. sensor detects obstacles and provides instant audio instructions to blind people.
Hardware Costs	The system comprises different hardware components that might be a little expensive. However, it remains one of the most reliable systems.

**Table 8:** Properties of Voice and Vibration Navigation Tools.

Voice and Vibration-Based Navigation System	
Better Obstacle Detection	The ultrasonic sensor accurately detects any obstacles in range of a visually impaired person.
Fast and Reliable Alerts	The ultrasonic sensor provides better navigation with voice and vibration feedback providing proper guidance.
Multipurpose	The system can be used for both indoor and outdoor environments.

**Table 9:** Properties of RGB-D Sensor-Based Navigation Tools.

RGB-D Sensor-Based System	
RGB Sensor	RGB sensor facilitates all the visually impaired with color sensing and object detection.
Camera-Based System	The RGB-D camera is used to support range-based floor segmentation.
Expensive	The multiple hardware components, including RGB-D sensors, make it an expensive option for blind people.

**Table 10:** Properties of Cellular Network-Based Navigation Tools.

Cellular Network-Based System	
Cellular Based Approach	Mobile phone communication makes this system work by using cellular towers to define the location.
Good for Indoors	The system works better in indoor environments.
Poor Positioning	The positioning is not very accurate due to the large signal ranges of cellular towers.

**Table 11:** Properties of Bluetooth-Based Navigation Tools.

Bluetooth-Based Approach	
Bluetooth-Based	The system mainly relies on Bluetooth networks to stay precise.
Not Expensive	The system is cheap as it relies on a Bluetooth network using pre-installed transmitters.
3D Indoor System	An indoor navigation system proposed by Cruz Ramos is also based on Bluetooth.
